# Production of Infectious Dengue Virus in *Aedes aegypti* Is Dependent on the Ubiquitin Proteasome Pathway

**DOI:** 10.1371/journal.pntd.0004227

**Published:** 2015-11-13

**Authors:** Milly M. Choy, October M. Sessions, Duane J. Gubler, Eng Eong Ooi

**Affiliations:** 1 Program in Emerging Infectious Diseases, Duke-National University of Singapore Graduate Medical School, Singapore, Singapore; 2 Interdisciplinary Research Group in Infectious Diseases, Singapore-MIT Alliance for Research and Technology, Singapore, Singapore; University of California, Berkeley, UNITED STATES

## Abstract

Dengue virus (DENV) relies on host factors to complete its life cycle in its mosquito host for subsequent transmission to humans. DENV first establishes infection in the midgut of *Aedes aegypti* and spreads to various mosquito organs for lifelong infection. Curiously, studies have shown that infectious DENV titers peak and decrease thereafter in the midgut despite relatively stable viral genome levels. However, the mechanisms that regulate this decoupling of infectious virion production from viral RNA replication have never been determined. We show here that the ubiquitin proteasome pathway (UPP) plays an important role in regulating infectious DENV production. Using RNA interference studies, we show *in vivo* that knockdown of selected UPP components reduced infectious virus production without altering viral RNA replication in the midgut. Furthermore, this decoupling effect could also be observed after RNAi knockdown in the head/thorax of the mosquito, which otherwise showed direct correlation between infectious DENV titer and viral RNA levels. The dependence on the UPP for successful DENV production is further reinforced by the observed up-regulation of key UPP molecules upon DENV infection that overcome the relatively low expression of these genes after a blood meal. Collectively, our findings indicate an important role for the UPP in regulating DENV production in the mosquito vector.

## Author Summary

DENV is a re-emerging mosquito-borne flavivirus and the lack of sustainable preventative or therapeutic measures renders it a significant health burden globally. Although suppression of mosquito populations represents the most widely used dengue control strategy, there has been limited efficacy with this method. Blocking host factors required for DENV replication in mosquitoes may thus serve as an effective anti-transmission strategy. We have recently observed that the UPP plays a critical role in regulating DENV egress from infected cells, but how the UPP contributes to DENV life cycle in mosquitoes remain ill-defined. We show here that the *Aedes aegypti* midgut has evolved to control persistent DENV infection by differentially regulating key genes in the UPP, without harm to itself. Using RNAi, knockdown of proteasome subunits, β1, β2 and β5, as well as other UPP-specific genes *in vivo* reduced the production of infectious virus without altering viral RNA replication in the mosquito. Targeting these host factors via dsRNA-mediated or chemical inactivation in the mosquito salivary glands may serve as a viable anti-dengue transmission strategy from mosquitoes to humans.

## Introduction

Dengue is the most important arthropod-borne viral disease globally. Dengue virus (DENV) propagates itself through cyclic human-mosquito-human transmission with *Aedes aegypti* (*Ae*. aegypti) being the principal vector [1]. The global distribution of the four antigenically distinct DENV (DENV-1, DENV-2, DENV-3 and DENV-4) along with their mosquito vectors causes an estimated 390 million infections annually [2]. Another 3 billion people that live in or travel to the tropics are at constant risk of infection with any of the four DENV serotypes [2]. Without an efficient vaccine to eliminate DENV transmission [3,4], vector control remains an integral part of any public health measure against dengue.

The effectiveness of vector control alone in preventing DENV transmission has had mixed success. While the eradication effort of *Ae*. *aegypti* by Pan American Health Organization (PAHO) in South America, and the vector control programs by Cuba and Singapore have had remarkable outcomes in reducing dengue incidence, their effectiveness have been temporary for reasons previously reviewed [5–8]. One major problem is the lowering of human herd immunity levels that comes with vector control [9]. The resultant increase in the proportion of the population susceptible to DENV infection necessitates an even lower vector population to prevent periodic epidemics [6]. Vector population suppression alone thus has a moving target. Better tools to either reduce vector population density or their vector competence are thus urgently needed to augment current preventive efforts.

Due to the limited coding capacity of its ~10.7kb genome [10], DENV is fully reliant on the host machinery for many, if not all, stages of the viral life cycle in the vertebrate host as well as mosquito vector. DENV host factors thus represent in humans, potential antiviral targets and, in mosquitoes, potential DENV transmission-blocking targets that could be developed into novel mosquito-based dengue control strategies.

In DENV-infected patients and mammalian cell lines, several studies have identified genes and pathways essential for DENV production and replication. One pathway that has been highlighted consistently is the ubiquitin proteasome pathway (UPP). The UPP is a major extra-lysosomal pathway for regulated protein degradation, clearing misfolded or obsolete proteins and maintaining protein homeostasis. The proteasome is the main driver of the UPP as it recognizes and degrades polyubiquitylated proteins, modified via covalent attachment of ubiquitin through the sequential activities of E1-activating, E2-conjugating, and E3 ligase enzymes [11]. A profound difference in gene expression and protein levels of key components of the UPP has been detected in DENV-infected cell lines and patients [12,13]. Concordant with this, both large-scale siRNA-screens identified components of the UPP as flaviviral replication promoting factors [14,15]. Pharmacological inhibition of the UPP, such as proteasome inhibition [12] or interference with the ubiquitin E1 activity [13] led to a significant reduction in DENV production although the underlying molecular mechanism was not identified.

In *Ae*. *aegypti*, transcriptome analyses have also identified the UPP to be essential for DENV replication and transmission [16–19]. Although UPP-specific genes such as *TSG101* (*AAEL012515*), *NEDD4* (*AAEL002536*) and SCF ubiquitin ligase (*AAEL004691*) have been shown to be modulated in response to DENV infection [17,20,21], the role of UPP in DENV replication in adult mosquitoes, as in humans, remains incompletely known. Here, we show that DENV requires the UPP to complete its lifecycle in *Ae*. *aegypti*. Although the mechanisms involved remains to be definitively determined, our findings provide a plausible explanation on how infectious DENV production is reduced despite persistent viral RNA levels in the mosquito midgut [22].

## Results

### Functional UPP is required for infectious DENV2 production in mosquito midgut

The role of the UPP in DENV infection was first investigated in the midgut of *Ae*. *aegypti*. DENV first establishes infection in the midgut of the female mosquito after a viremic blood meal. It then spreads systemically to the other organs, such as the head and salivary glands of the mosquito to establish persistent, lifelong infection [23]. Continued production of DENV in the salivary glands enables transmission of virus to new susceptible human hosts and thus ensures the survival of DENV. In the midgut, however, infectious DENV titers and viral antigen decrease after their peak at 7–10 days post blood meal (dpbm) despite continued replication of its RNA genome [22]. The mechanism that regulates this outcome is unknown.

**Fig 1:**
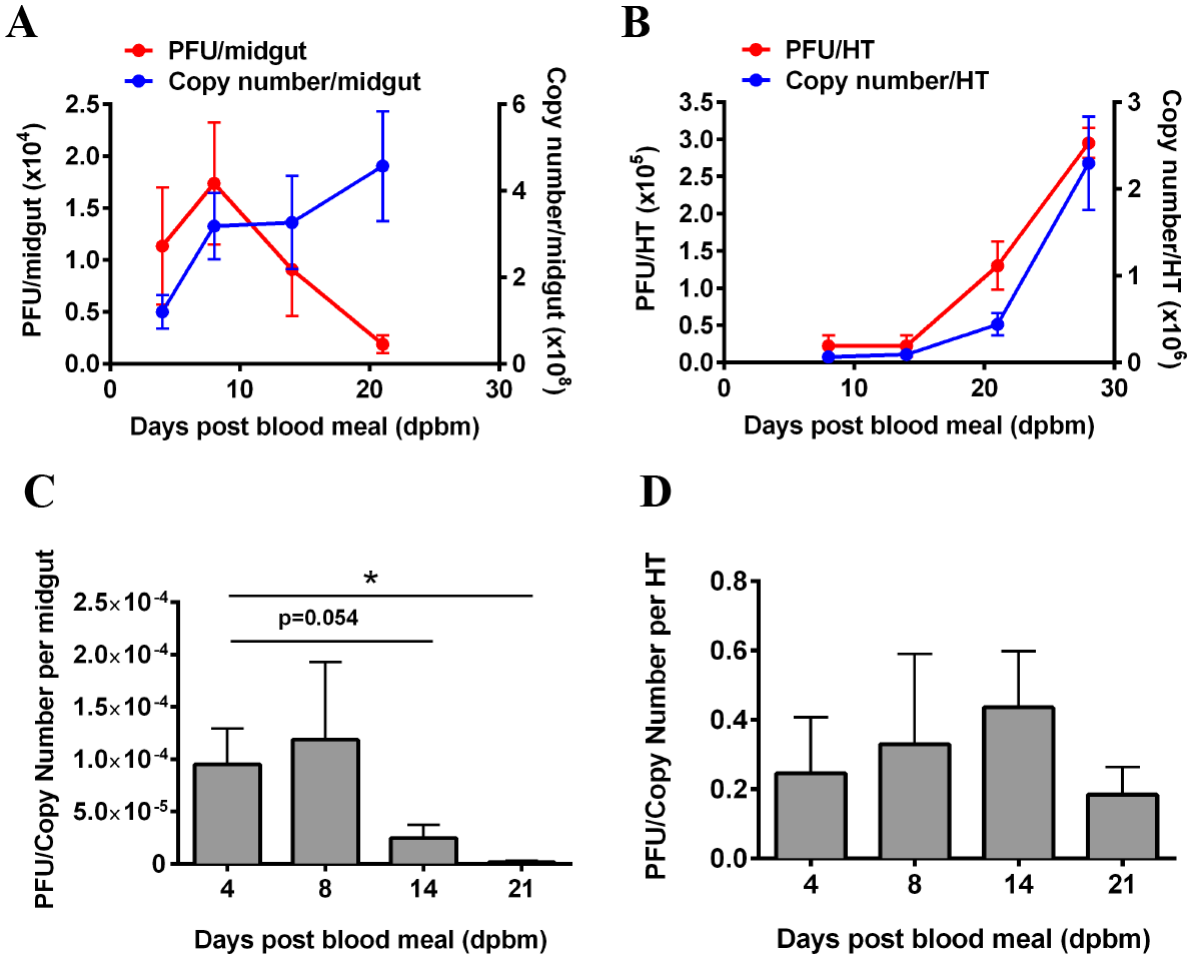
Characterization of DENV-2 replication in the midguts and heads/thoraces of *Ae*. *aegypti* following ingestion of an infectious blood meal. (A) In the midgut, viral titers increased linearly until 8 dpbm and declined thereafter. In contrast, viral RNA remained stable between 8 to 21 dpbm. Mean ± SEM, N = 8–10. (B) In the heads/thoraces (HT), the increase in both infectious particles and viral RNA are positively correlated over time. Viral RNA copy number increases with increasing viral titers. Mean ± SEM, N = 8–10. (C-D) A corresponding decrease in PFU/Copy number was observed in the midgut over time, with no significant change in the head/thorax (HT).

We first tested if the observation described previously [22] could be replicated in our hands by characterizing virus replication in the midgut of *Ae*. *aegypti* following ingestion of blood spiked with DENV2. DENV2 infection was detected in midgut epithelial cells as early as 2 dpbm, peaked at 8 dpbm and decreased thereafter (Fig 1A). The amount of viral antigen detected using immunofluorescence decreased starting at 10 dpbm as well (S1 Fig). However, measurement of DENV2 RNA using qRT-PCR revealed no significant reduction in the viral RNA copy number as late as 21 dpbm (Fig 1A). In contrast, both DENV2 titers and viral RNA copy number in the head/thorax were positively correlated through to the limit of the lifespan of *Ae*. *aegypti* in the laboratory (Fig 1B). Correspondingly, the ratio of PFU to RNA copy number decreased significantly over time in the midgut but not in the head/thorax (Fig 1C and 1D). This observation recapitulated previously reported findings [22] and guides our experimental design to study proteasome function 8 days post infection (dpi).

**Fig 2:**
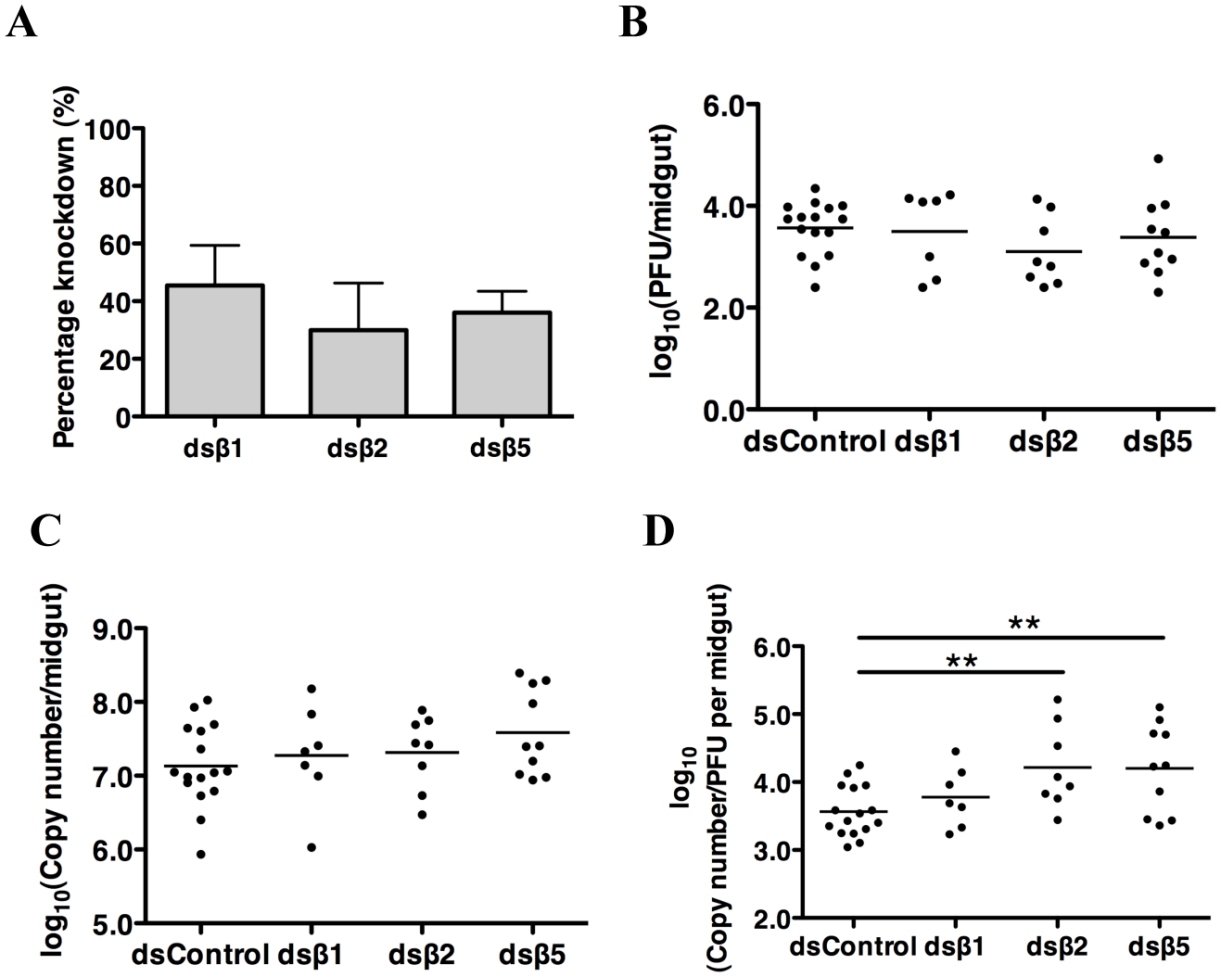
Proteasome inhibition of β2 and β5 subunits decouples infectious DENV-2 production from viral RNA replication in mosquito midguts. (A) Silencing efficiencies of β subunits of the proteasome were determined by gene specific qPCR, and expression values were normalized against dsRNA control targeting random sequences from pGEM T easy vector 10 days after dsRNA inoculation. N = 10. (B) No statistically significant difference was observed in virus titer per midgut at 6 dpbm after knockdown of β1, β2 and β5 subunits. Mean ± SEM, N = 7–16. (C) No statistically significant differences were observed in DENV2 viral RNA levels per midgut 6 dpbm after β1, β2 and β5 subunits knockdown. Mean ± SEM, N = 7–16. (D) log(PFU/Copy Number) was significantly lower after β2 and β5 knockdown. Mean ± SEM, N = 20–22. Student’s t test, **p<0.01.

To demonstrate a functional requirement of proteasome on the virus life cycle besides viral entry, RNA interference (RNAi)-mediated gene silencing of the catalytic subunits of the proteasome, β1 (capase-like activity), β2 (trypsin-like activity) and β5 (chymotrypsin-like activity), was performed *in vivo* as described elsewhere [24]. Female mosquitoes were orally infected with DENV2 before inoculation of dsRNA at 3 dpbm to preclude the possibility that knockdown of these genes could interfere with endocytosis and hence DENV entry in the midgut epithelial cells [14,25]. DENV2 infected mosquitoes inoculated with dsRNA targeting random sequences from pGEM T easy vector served as a control for these experiments. The mosquito midguts were then harvested and analyzed at 6 dpbm, two days before the decline in infectious DENV2. Efficacy of RNAi-mediated knockdown was assessed by gene-specific qRT-PCR (Fig 2A) and the effect of their knockdown on viral propagation was measured by both plaque assay and DENV-specific qRT-PCR. Knockdown of the β1, β2 and β5 subunits of the proteasome significantly reduced the proportions of infected mosquitoes compared to mosquitoes inoculated with control dsRNA (Table 1). Among those mosquitoes that were infected, differences in plaque titers (Fig 2B) and viral genomic RNA copies (Fig 2C) were not statistically significant although the ratio of PFU to RNA copy number per midgut decreased significantly after β2 and β5 knockdown (Fig 2D). These findings indicate that a functional proteasome is essential for DENV to establish a successful infection in the mosquito midgut.

**Table 1:** Percentage of DENV2-infected mosquitoes after knockdown of proteasome subunits (p value; Fischer’s Exact Test).

	Percentage with infectious particles detected (%)	Infected/Total (%)	p-value
Virus Control	83.3	10/12 (83.3)	-
dsControl	80.0	16/20 (80.0)	-
dsβ1	31.8	7/22 (31.8)	0.0023
dsβ2	40.0	8/20 (40.0)	0.0225
dsβ5	45.8	10/22 (45.8)	0.0289

### Regulation of UPP-specific genes decouples infectious DENV2 production from viral RNA replication in mosquito midguts

**Fig 3:**
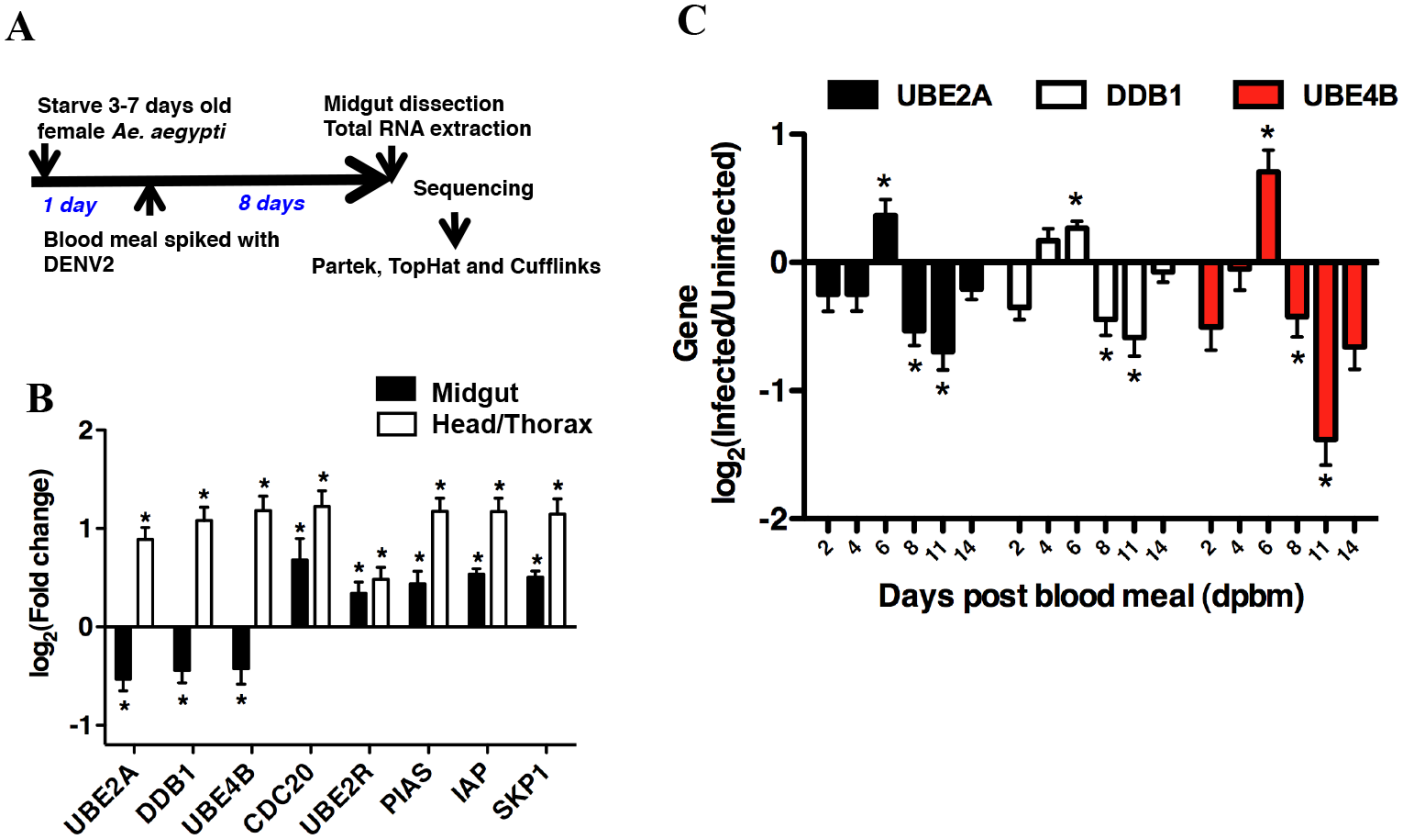
Organ- and time-specific regulation of genes belonging to the UPP in response to DENV infection. (A) Experimental workflow of transcriptome analysis of *Ae*. *aegypti* midgut 8 dpbm. (B) Validation of differentially regulated UPP-specific genes using qRT-PCR. Expression levels of UPP genes in midgut 8 dpbm and head/thorax 21 dpbm were compared. Contrasting expression levels of *UBE2A*, *DDB1* and *UBE4B* were observed, with these genes being down-regulated in the midgut, but up-regulated in the head/thorax when compared with uninfected midguts or heads/thoraces respectively. Mean ± SEM, N = 12. Student’s t test, *p < 0.05. (C) Gene expression levels in individual infected midguts were measured using qRT-PCR, normalized to GAPDH and compared to midguts from uninfected blood fed mosquitoes. Mean ± SEM. N = 12–16. Student’s t test, *p < 0.05.

The above observations also suggest that differential regulation of the proteasome or other genes in the UPP could be involved in the reduction of infectious DENV2 titers observed naturally in the midgut at 8 dpbm. To test this, we utilized RNAseq to analyze the transcripts of the female *Ae*. *aegypti* midgut at 8 dpbm. This approach overcomes the current scarcity in mosquito protein-specific reagents as well as difficulty in designing oligonucleotide primers that accurately complement outbred, field-collected mosquitoes supplemented monthly (10%) to the *Ae*. *aegypti* colony in our insectary. The sequence obtained would then guide subsequent primer design for qPCR measurement of specific mRNA transcripts. The experimental workflow is depicted in Fig 3A. Mosquitoes that were fed on blood without spiked DENV2 served as control. RNAseq analysis was performed for a pool of 100 dissected DENV-infected midguts and compared to a similarly pooled blood fed control using Cufflinks v13.0 [26]. The quality of our libraries and sequencing performance was assessed using Partek Genomic Suite v6.6 (Partek Incorporated) (S1 Table).

Our results showed no differential regulation in any components of the proteasome. Instead, several genes within the UPP were found to be differentially regulated (S2 Fig). A subset of these was validated by qRT-PCR in a separate experiment using individual midguts from mosquitoes fed with the same blood meal without or spiked with DENV2 (Fig 3B). Interestingly, 3 genes were down-regulated in the midgut but significantly up-regulated in the head/thorax at 21 dpbm (Fig 3B), when the virus is replicating actively, before reaching a peak (Fig 1B). These observations suggest that up-regulation of the 3 UPP-related genes; *UBE2A* (*AAEL002118*), *DDB1* (*AAEL002407*) and *UBE4B* (*AAEL006910*) are needed for DENV2 to complete its life cycle in the midgut.

If expression of these 3 UPP-related genes were required for DENV production, then it follows that these genes must be up-regulated prior to the peak of virus replication at 8 dpbm, to allow for systemic spread of DENV to the salivary glands before their expression is down-regulated. We thus examined the kinetics of the expression of these 3 genes. Expression levels of these 3 UPP genes were measured at 2, 4, 6, 8, 11 and 14 dpbm. Indeed, all 3 genes were significantly up-regulated at 6 dpbm compared to blood-fed uninfected control, but were all down-regulated from 8 dpbm onwards (Fig 3C).

**Fig 4:**
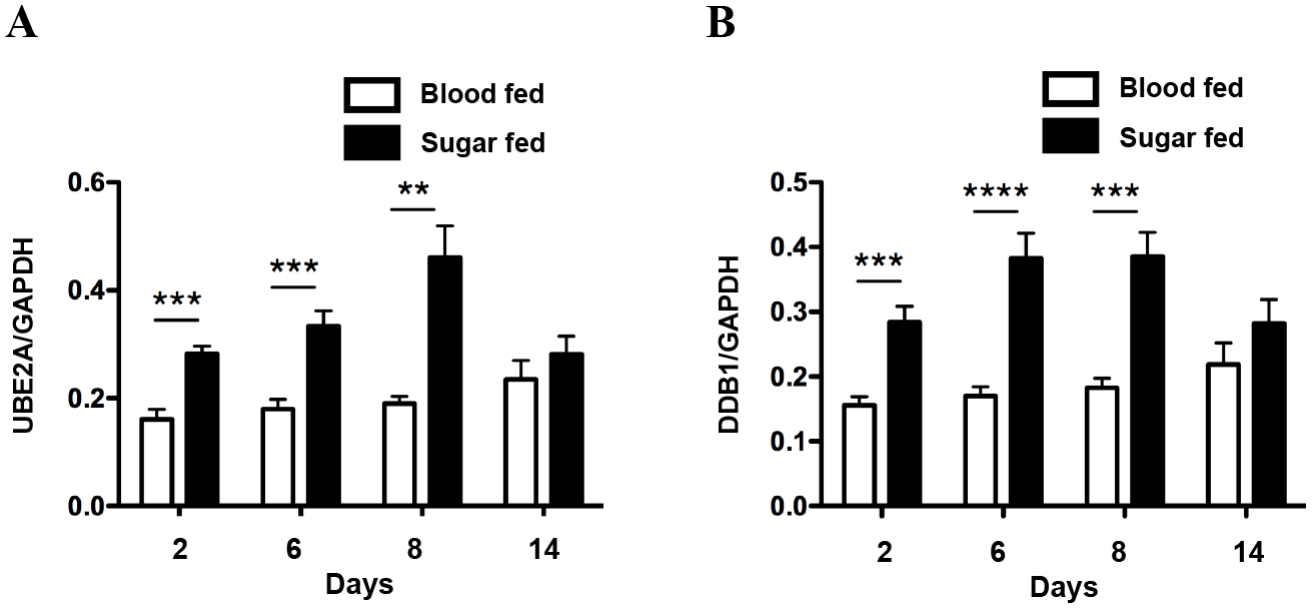
Ingestion of blood meal does not modulate gene expression of *UBE2A* and *DDB1*. Sugar fed and blood fed mosquitoes were harvested at different time-points and individual midguts were dissected for analyses. Gene expression levels for (A) *UBE2A* and (B) *DDB1* were measured using qRT-PCR and normalized to GAPDH. Expression levels of *UBE2A* and *DDB1* in blood fed mosquitoes remained consistently unchanged over the course of 14 days, whereas ingestion of a sugar meal increases expression levels of *UBE2A* and *DDB1* significantly until 8 days relative to the blood fed mosquitoes. Mean ± SEM. N = 12. Student’s t test, **p < 0.01, ***p<0.001, ****p<0.0001.

Next, we asked if the expression differences in these genes are mediated by a blood meal or DENV infection. Indeed, ingestion of a blood meal has been correlated with an enhancement of mosquito genes involved in digestive activity and a suppression of genes involved in environmental stimuli perception and innate immunity [27]. To test this, we compared the expression levels of *UBE2A* and *DDB1* in uninfected sugar fed and uninfected blood fed mosquitoes in a time course study. Our data indicates that ingestion of a sugar meal increases the expression levels of *UBE2A* and *DDB1* significantly relative to the blood fed mosquitoes. Moreover, the expression levels of *UBE2A* and *DDB1* in blood fed mosquitoes remained consistently unchanged over the course of 14 days (Fig 4A and 4B). This suggests that the transient up-regulation of *UBE2A* and *DDB1* observed after dengue infection at 4 to 6 dpbm may be mediated, at least in part, by a mechanism encoded in the DENV genome to enable the virus to complete its life cycle in the midgut before systemic spread to the salivary glands.

**Fig 5:**
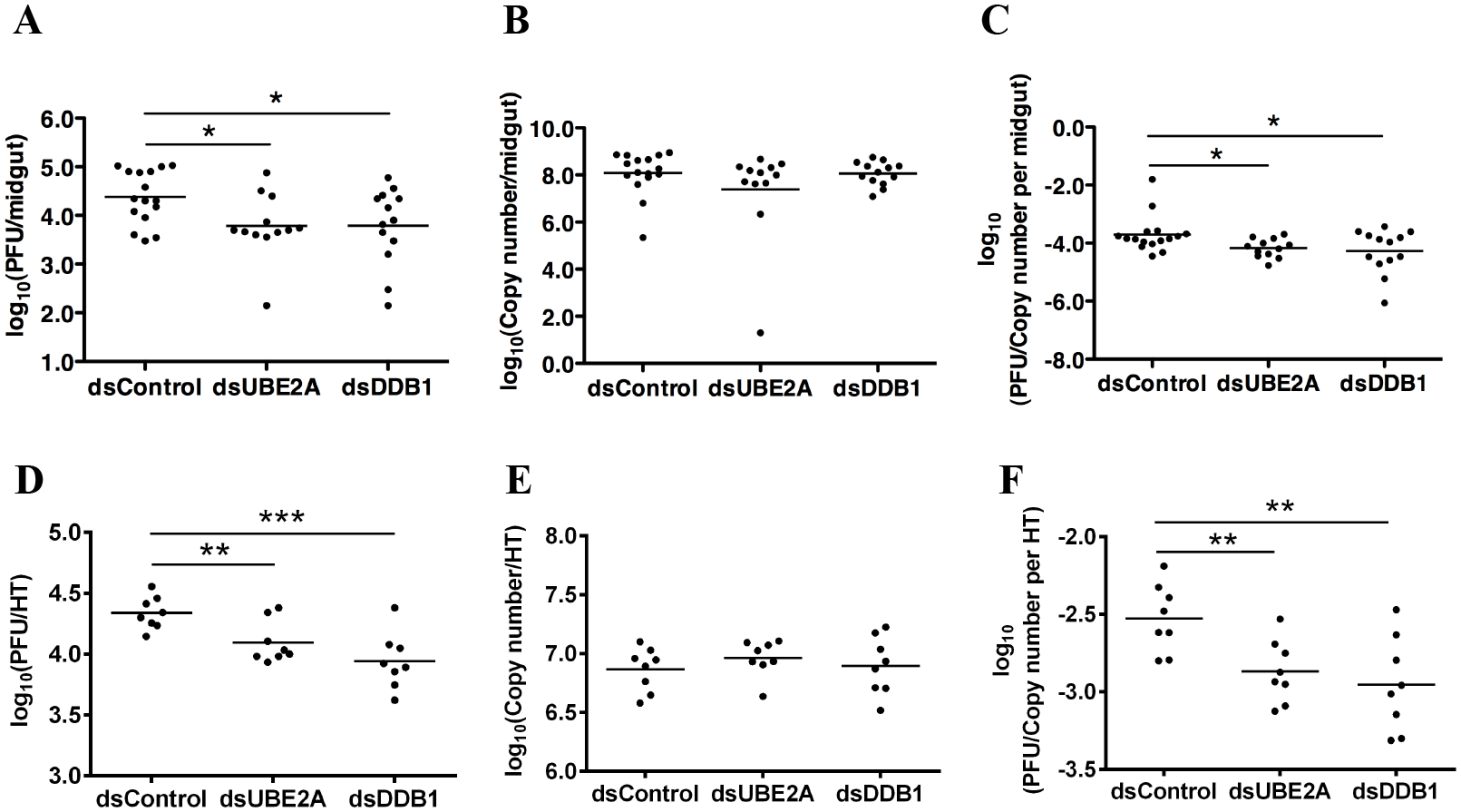
Knockdown of UBE2A and DDB1 decouples infectious DENV2 production from viral RNA replication in mosquitoes. (A) In the infected midguts, virus titers declined significantly after knockdown of UBE2A and DDB1 at 6 dpbm. N = 12–16. Student’s t test, *p < 0.05. (B) In the infected midguts, no statistically significant differences were observed in DENV2 viral RNA levels 6 dpbm after gene knockdown. N = 12–16. (C) Ratio of midgut infectious titers to viral RNA levels 6 dpbm after gene knockdown. N = 12–16. Student’s t test, *p < 0.05. (D) In infected heads/thoraces (HT), virus titers at 8 days post intra-thoracic inoculation declined significantly after knockdown of UBE2A and DDB1. N = 8–10. Student’s t test, **p < 0.01, ***p<0.001. (E) In infected heads/thoraces (HT), no statistically significant differences were observed in DENV2 viral RNA levels after gene knockdown. N = 8–10. (F) Ratio of head/thorax (HT) infectious titers to viral RNA levels after gene knockdown 8 dpi. N = 12–16. Student’s t test, **p<0.01.

To demonstrate a functional requirement of *UBE2A*, *DDB1* and *UBE4B* on DENV production in the mosquito, RNAi-mediated gene silencing was performed. As a negative control, gene silencing was also performed for *UBE2M* (*AAEL009026*), which was not detected in our RNAseq analysis. Efficacy of RNAi-mediated knockdown was assessed by gene-specific qRT-PCR (S3A Fig). Compared to the infected blood fed dsRNA control, knockdown of *UBE2A* and *DDB1* in infected midguts resulted in a significant reduction of infectious DENV2 titers (Fig 5A) but not viral genomic RNA (Fig 5B). Correspondingly, ratio of PFU to RNA copy number per midgut decreased significantly (Fig 5C). No significant differences were observed for *UBE4B* or *UBE2M* knockdown (S3B and S3C Fig).

To further validate the function of *UBE2A* and *DDB1* in the DENV life cycle, we tested if silencing of these two genes would also reduce plaque titers without altering RNA replication in the head/thorax of *Ae*. *aegypti*. Mosquitoes were infected via intra-thoracic inoculation to ensure that all mosquitoes have disseminated infections by bypassing the midgut. At 3 dpi, dsRNA was inoculated into the thorax. Heads/thoraces of the mosquitoes were harvested 8 dpi. As expected, knockdown of *UBE2A* and *DDB1* reproduced the decoupling of infectious virus production from RNA replication; infectious DENV2 titers decreased (Fig 5D) but not DENV2 RNA levels (Fig 5E). Correspondingly, the ratio of PFU to RNA copy number per head/thorax decreased significantly (Fig 5F). Collectively, these observations indicate a functional role for the UPP in regulating DENV production in the mosquito vector.

## Discussion

The role of the UPP in the DENV life cycle in the mosquito vector is incompletely understood. Using RNAi mediated gene silencing, our data suggests that components of the UPP are critical for infectious DENV production in the mosquito midgut. Reduced expression of these genes resulted in decreased infectious virus titers, albeit with no effect on DENV genome replication. This is concordant with previous studies in mosquitoes where various UPP-specific genes such as *TSG101* (AAEL012515), *NEDD4* (AAEL002536) and SCF ubiquitin ligase (AAEL004691) have also been identified as potential host factors for DENV replication [17,20,21,28]. Due to genetic variability across various mosquito strains and DENV-2 strains, as well as variation in the methodology of the experiments such as data analysis and time-points used, it is not surprising that the UPP-specific genes detected in these studies were not the same individual genes. As these molecules function to signal for the activation of the effector of the UPP, the proteasome, we showed via functional knockdown of the proteasome that DENV may depend on the pathway rather than signaling intermediates for successful completion of its life cycle in mosquitoes.

Intriguingly, the dependence on the UPP by DENV to complete its life cycle is further reinforced by the possibility that the up-regulation of key UPP molecules is triggered by infection to overcome the relatively low expression of these genes after a blood meal. Both *UBE2A* and *DDB1* act upstream of the proteasome in the UPP; the former belonging to the E2 ubiquitin-conjugating enzyme family, and the latter functioning as an adaptor molecule for the cullin 4-ubiquitin E3 ligase complex [29]. Teasing apart how DENV, or other host response to infection, mediates this change in *UBE2A* and *DDB1* expression would be interesting.

How inhibition of the UPP reduces infection rate and decouples viral RNA replication from infectious virus production in the mosquito midgut remains to be definitively determined. A functional UPP has been shown to be required for DENV entry into cells through endocytosis although this effect appears to be cell specific [14,30]. Indeed, as partial knock-down of the proteasome components was performed 3 days after an infected blood meal, one plausible explanation for the reduced infection rate could be that subsequent entry of DENV into adjacent cells are inhibited, thereby reducing the extent of infection in the midgut.

Besides, endocytosis, proper egress of several viruses from infected cells for subsequent rounds of infection has also been shown to require a functional UPP. This notion is supported by studies on retroviruses demonstrating that disruption of the proteasome function depletes the free ubiquitin pool [31], which is necessary for the ubiquitylation of late domain on Gag protein for proper viral budding [32,33]. It is also possible that a functional UPP is needed to nullify inhibitors of excretory vesicles for successful egress, thereby reducing the amount of infectious DENV released from infected cells. UBE2A targets several short-lived regulatory proteins for poly-ubiquitylation and subsequent turnover by the 26S proteasome [34]. DDB1 has been shown to facilitate the ubiquitylation and subsequent proteasome-mediated degradation of STATs for the *Rubulavirus* genus of *Paramyxoviridae* [35, 36]. This is in agreement with recent studies, which show that DENV can exploit the UPP to degrade host proteins that have antiviral activities. The NS5 protein co-opts UBR4, an E3 ubiquitin ligase, to evade the innate immune response by antagonizing type I interferon signaling [37] and hence the expression of interferon stimulated genes that might inhibit infectious DENV production.

The UPP is also an integral component of the unfolded protein response (UPR) that is triggered upon endoplasmic reticulum (ER) stress [38–40]. It is thus plausible that without the upregulation of the UPP during DENV infection, the increased protein production exacerbates ER stress in the midgut. ER stress is known to activate the eIF2α-mediated translational repression of cellular mRNA. The eIF2α protein is an effector of the PKR-like ER kinase (PERK) pathway in the UPR and phosphorylation of this protein prevents GDP-GTP exchange on eIF2α by the guanine nucleotide exchange factor eIF2B, thereby inhibiting recycling of the ternary complex that contains the initiator methionine Met-tRNAi [41–43]. Consequently, translation initiation is decreased which could lead to reduced production of viral proteins necessary for virion assembly thereby completing its life cycle. This explanation would be consistent with the observed reduction in viral titers and viral antigen despite continual increase in viral RNA copies after 8 dpbm.

Alternatively, ER stress could also lead to reduced translation of host proteins in the exocyst complex, which are critical for effective DENV egress via exocytosis (Choy et al., In Press). In monocytes, inhibition of the UPP did not prevent viral RNA or protein synthesis but rather resulted in accumulation of packaged virions within Golgi-derived vacuoles due to reduced fusion of post-Golgi vesicles to plasma membranes. This effect would then inhibit the spread of DENV to new susceptible cells in the midgut. Further studies will be needed to identify which of these possible mechanisms limit sustained production of infectious DENV from the midgut without altering viral RNA replication.

In conclusion, our study provides new insights into the role a functional UPP plays in DENV infection which may explain a long observed phenomenon in the mosquito midgut. Targeting the UPP in the salivary glands may serve as a viable anti-dengue transmission strategy in mosquitoes.

## Materials and Methods

### Mosquitoes

*Ae*. *aegypti* mosquitoes were obtained from a colony at the Duke-NUS Graduate Medical School. The colony was established in 2010 with specimens collected in Ang Mo Kio, Singapore, and supplemented monthly with field-collected mosquitoes (10% of colony) to maintain genetic diversity.

### Virus stock

DENV2 ST used in this study is a clinical isolate from Singapore. DENV2 ST was propagated in the Vero or C6/36 cell lines and harvested when 75% or more of the cells showed cytopathic effect. Infectious titer was determined by plaque assay. To obtain high virus titer, the virus was purified through 30% sucrose cushion as previously described [44]. Virus pellets were resuspended in 5 mM Hepes, 150 mM NaCl, and 0.1 mM EDTA (HNE) buffer and stored at −80°C until use. Infectious titer was determined by plaque assay.

### Plaque assay

Serial dilutions (10-fold) of virus were added to BHK-21 cells in 24-well plates and incubated for 1 hour at 37°C. Media was aspirated and replaced with 0.8% methyl-cellulose in maintenance medium (RPMI-1640, 2% FCS, 25 mM Hepes, penicillin, and streptomycin). After 5 days at 37°C, cells were fixed with 20% formaldehyde at room temperature for 20 min and washed with water, and 1 mL of 1% crystal violet was added for 20 min. The plates were washed and dried, and PFU/mL were calculated.

### DENV2 infection in mosquitoes

3- to 4-day-old mosquitoes were infected with DENV2 ST. For oral infection, purified DENV2 (1 x 10^9^ PFU/mL) was mixed 1:10 with commercially obtained pig blood. The blood meal was maintained at 37°C for 10 min prior to feeding 3- to 4-day-old mosquitoes using an artificial feeding system. Mosquitoes were cold anesthetized to pick only fully engorged mosquitoes to be used for subsequent experiments. Infectious blood meal titers were measured using plaque assay and ranged from 7.5–9 x 10^7^ PFU/mL. Intra-thoracic inoculation of DENV2 was performed as previously described [45,46].

### Immunofluorescence assay

Midguts were dissected in PBS and placed onto a Teflon coated glass slide, air dried and then fixed in 80% acetone for 10 min. The slide was rinsed with PBS and air-dried. Flavivirus cross-reactive 4G2 (HB112) (diluted 1:10) was used and incubated at 37°C for 45 min in a humidified chamber, and washed with PBS before drying. FITC-conjugated goat anti-mouse IgG (diluted 1:2000) was added and incubated at 37°C for 30 min in the humidified chamber and then washed with PBS. Slides were dried and mounted with buffered glycerol before imaging under a fluorescent microscope.

### Gene silencing assays

RNAi-mediated gene silencing in mosquitoes was performed as previously described [24]. Briefly, 3- to 4-day-old female mosquitoes infected either through ingestion of DENV2 spiked blood meal or inoculated intrathoracically with 100MID_50_ of DENV2 were held until 3 dpbm or dpi, at which time they were cold anesthetized and injected with 2 μg of dsRNA per mosquito. Orally infected mosquitoes injected with dsRNA containing random sequences from pGEM T easy vector were used as controls. Surviving mosquitoes were harvested 6 dpbm or 8 dpi after which the midguts or heads/thoraces of these mosquitoes were removed respectively, and individually homogenized in DMEM (supplemented with 10% FCS, penicillin and streptomycin) using a high-speed homogenizer FastPrep-24 (MP Biomedicals). Each homogenized sample was centrifuged and the supernatant was removed and stored at −80°C until they were titrated by plaque assay and qRT-PCR. The dsRNAs used were synthesized using the HiScribe T7 *in vitro* transcription kit (New England Biolabs). The primer sequences used for dsRNA synthesis and primer sequences used to confirm gene silencing by qRT-PCR are presented in S2 and S3 Tables respectively.

### Generation of whole-transcriptome cDNA library

DENV2-infected and uninfected blood fed control mosquitoes were dissected at 8 dpbm, and 100 midguts for each condition were pooled and stored in TRIzol reagent (Invitrogen). Total RNA from mosquito midguts was extracted using TRIzol and rRNA was removed by hybridization using RiboMinus Eukaryote Kit for RNA-Seq (Invitrogen). Polyadenylated mRNA was then isolated from mosquito midguts by one round of selection with the Dynabeads mRNA Purification Kit (Invitrogen). Quality of mRNA was assessed by electrophoresis on the Bioanalyzer 2100 (Agilent). For RNAseq sample preparation, NEBNext mRNA Sample Prep Master Mix Set 1 was used according to the manufacturer’s protocol (NEB). Briefly, 0.5ug mRNA was used for fragmentation and then subjected to cDNA synthesis using SuperScript III Reverse Transcriptase (Invitrogen) and random primers. The cDNA was further converted into double stranded cDNA and, after an end repair process (Klenow fragment, T4 polynucleotide kinase and T4 polymerase), was ligated to Illumina paired end (PE) adaptors. Size selection was performed using a 2% agarose gel, generating cDNA libraries ranging in size from 275–325bp. Finally, the libraries were enriched using 15 cycles of PCR and purified by the QIAquick PCR purification kit (Qiagen).

### RNAseq analysis

cDNA libraries were sequenced on Illumina HiSeq 2000 (Duke-NUS Genome Biology Facility, Singapore). Resulting reads were mapped to the AaegL1 library built from the *Aedes aegypti* genome [47] using Tophat v1.3.0 (http://tophat.cbcb.umd.edu/index.html) [48] with the coverage-search, microexon-search and butterfly-search options. Differential gene expression analysis was done using Cufflinks v1.3.0 (http://cufflinks.cbcb.umd.edu/) [26] with the multi-read-correct (Cufflinks), -r and -s (Cuffcompare; using the same annotation gtf and AaegL1 fasta files respectively as in Tophat) and the frag-bias-correct (same AaegL1 fasta file used for Tophat) and multi-read-correct options.

### Statistical analysis

All calculations were done using GraphPad Prism v5.0 (GraphPad Software Inc.).

## Supporting Information

S1 Fig Detection of viral antigen after DENV infection of *Aedes aegypti*. Midguts dissected at different time points (N = 5) were assayed by immunofluorescence assay to detect DENV viral antigen (green). At each time point, (A) 2 dpbm, (B) 8 dpbm, (C) 10 dpbm, (D) 15 dpbm; a representative midgut is presented. The amount of viral antigen detected using immunofluorescence increased until 8 dpbm and subsequently decreased around 10 dpbm. Magnification = 400×.
(TIFF)

S2 Fig RNA-sequencing of *Ae*. *aegypti* midgut. Differentially regulated genes (red for down-regulation, blue for up-regulation) belonging to the UPP in KEGG pathway (*Ae*. *aegypti*). P-value is lesser than the FDR < 0.1 after Benjamini-Hochberg correction for multiple-testing.
(TIF)

S3 Fig Functional analysis of UPP-specific genes in individual midguts of *Ae*. *aegypti*. (A) Silencing efficiencies of UPP-specific genes were determined by gene-specific qPCR, and expression values were normalized against control. Mean ± SEM. N = 7–8. (B) Candidate genes were silenced in DENV2-infected mosquitoes, and midgut virus titers at 6 days post blood meal were determined by plaque assay. No statistically significant differences were observed in virus titers after gene knockdown. N = 7–8. (C) No statistically significant differences were observed in DENV2 viral RNA after gene knockdown. N = 7–8.
(TIF)

S1 Table Summary of Illumina HighSeq 2000 RNA-sequencing using Partek Genomic Suite v6.6. (PDF)

S2 Table Primers for qPCR (primer combination can be used for either RNAi verification or gene expression). (PDF)

S3 Table Primers for RNAi assays. (PDF)
