# Supplementary figures and images for: Production of Infectious Dengue Virus in Aedes aegypti Is Dependent on the Ubiquitin Proteasome Pathway
Source: PLoS Negl Trop Dis. 2015 Nov 13;9(11):e0004227. doi: 10.1371/journal.pntd.0004227 (PMC4643912; doi:10.1371/journal.pntd.0004227)

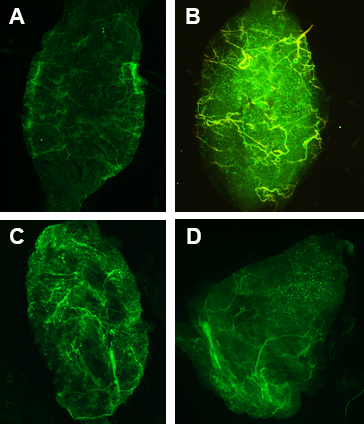

Supplement: S1 Fig — Midguts dissected at different time points (N = 5) were assayed by immunofluorescence assay to detect DENV viral antigen (green). At each time point, (A) 2 dpbm, (B) 8 dpbm, (C) 10 dpbm, (D) 15 dpbm; a representative midgut is presented. The amount of viral antigen detected using immunofluorescence increased until 8 dpbm and subsequently decreased around 10 dpbm. Magnification = 400×. (TIFF) [file pntd.0004227.s001.tiff]

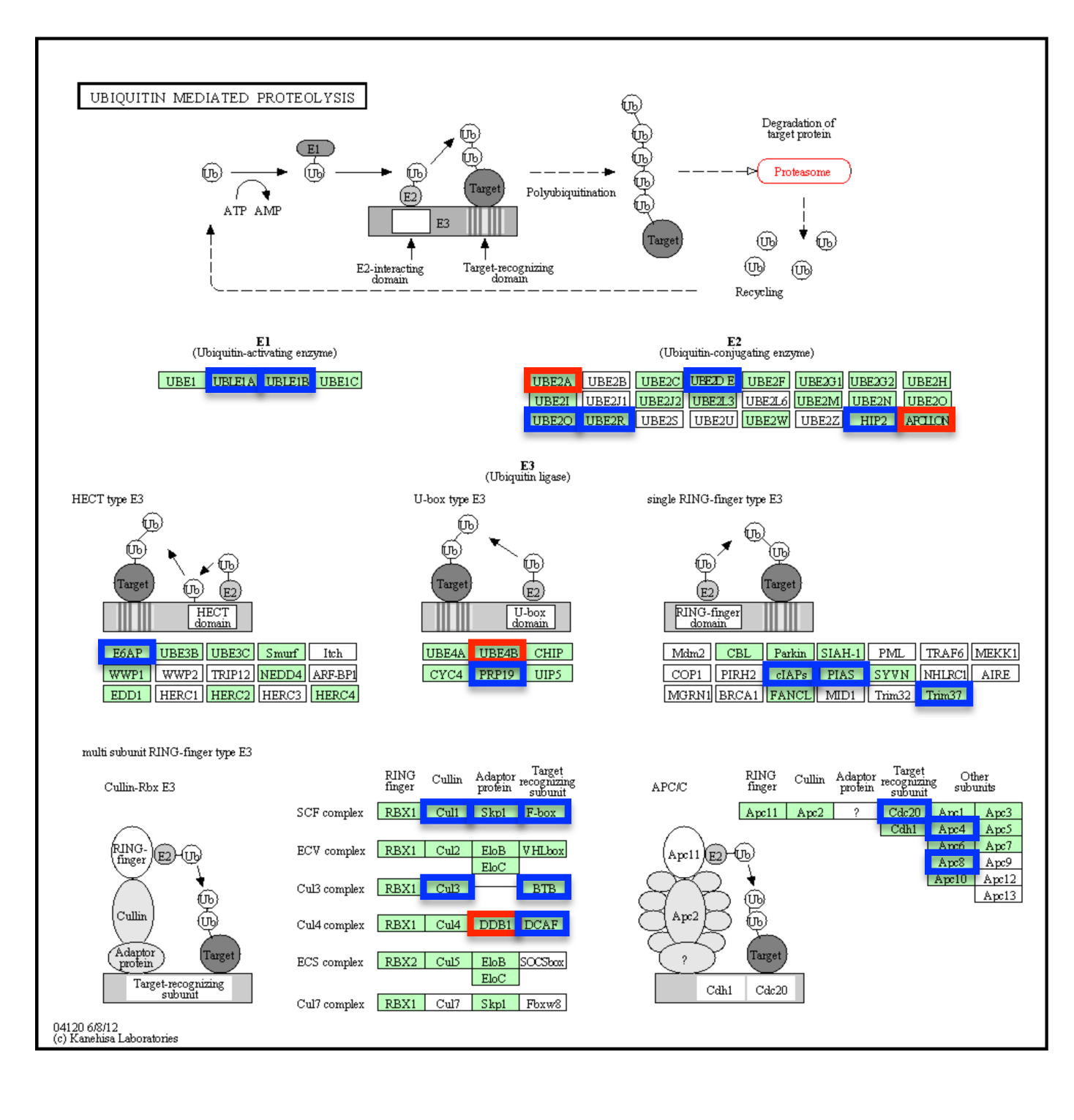

Supplement: S2 Fig — Differentially regulated genes (red for down-regulation, blue for up-regulation) belonging to the UPP in KEGG pathway (Ae. aegypti). P-value is lesser than the FDR < 0.1 after Benjamini-Hochberg correction for multiple-testing. (TIF) [file pntd.0004227.s002.tif]

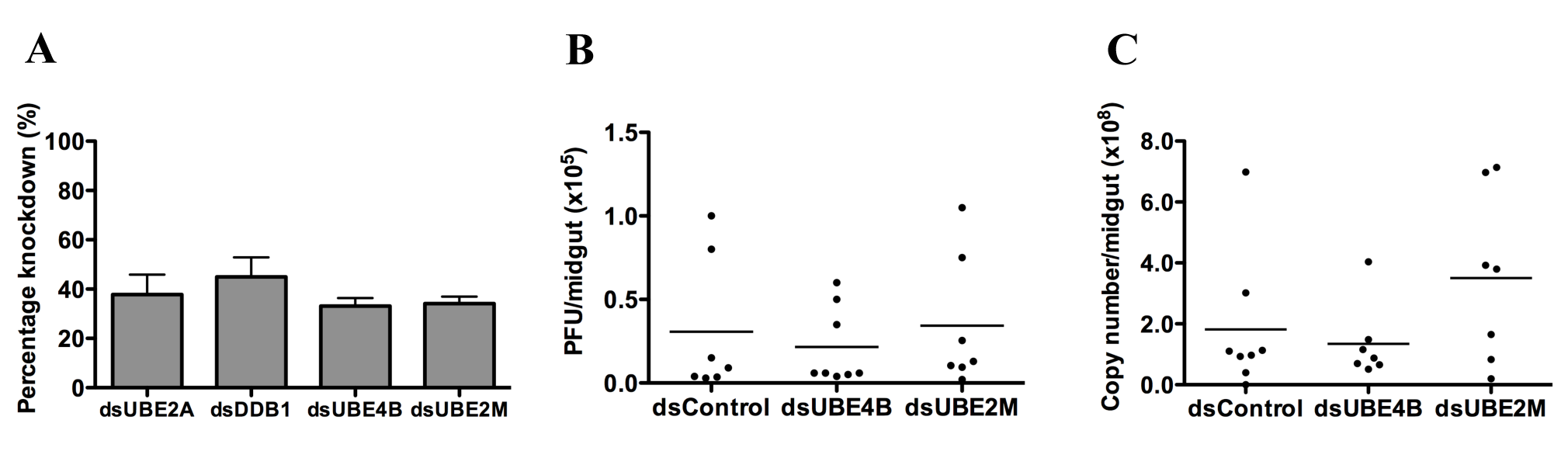

Supplement: S3 Fig — (A) Silencing efficiencies of UPP-specific genes were determined by gene-specific qPCR, and expression values were normalized against control. Mean ± SEM. N = 7–8. (B) Candidate genes were silenced in DENV2-infected mosquitoes, and midgut virus titers at 6 days post blood meal were determined by plaque assay. No statistically significant differences were observed in virus titers after gene knockdown. N = 7–8. (C) No statistically significant differences were observed in DENV2 viral RNA after gene knockdown. N = 7–8. (TIF) [file pntd.0004227.s003.tif]
