# Supplementary material for: Production of Infectious Dengue Virus in Aedes aegypti Is Dependent on the Ubiquitin Proteasome Pathway
Source: PLoS Negl Trop Dis. 2015 Nov 13;9(11):e0004227. doi: 10.1371/journal.pntd.0004227 (PMC4643912; doi:10.1371/journal.pntd.0004227)
Supplement: S1 Table — (PDF) [file pntd.0004227.s004.pdf]

S1 Table. Summary of Illumina HighSeq 2000 RNA-sequencing using Partek Genomic Suite v6.6

Sample ID	Number of Alignments	Total Number of Reads	Percentage of reads which fully overlap exon	Percentage of transcripts with reads	Total reads with junctions	Reads with junctions that are compatible with a transcript
**DENV2ST**	32567237	15880324	67.3503	81.8814	2202876	1716335
**Uninfected**	11994298	4676021	55.1425	67.906	346293	245503
