# Supplementary material for: Production of Infectious Dengue Virus in Aedes aegypti Is Dependent on the Ubiquitin Proteasome Pathway
Source: PLoS Negl Trop Dis. 2015 Nov 13;9(11):e0004227. doi: 10.1371/journal.pntd.0004227 (PMC4643912; doi:10.1371/journal.pntd.0004227)
Supplement: S2 Table — (PDF) [file pntd.0004227.s005.pdf]

S2 Table. Primers for qPCR (primer combination can be used for either RNAi verification or gene expression).

Gene	Forward Primer (5' to 3')	Reverse Primer (5' to 3')
**DEN2 Envelope**	CAGGTTATGGCACTGTCACGAT	CCATCTGCAGCAACACCATCTC
**DEN2 3'UTR**	TTGAGTAAACYRTGCTGCCTGTAGCTC	GAGACAGCAGGATCTCTGGTCTYTC
**Mosquito GAPDH**	CGCTTCCTGTACCACCAACTGC	CCATCACGCCACAGCTTACCAG
**Mosquito UBE2A**	TCTTCGAGTTGTGGCCTCTT	CCTTGGAGCTGTTCAGGAAG
**Mosquito DDB1**	GTGGAATTCAACGGACGAGT	AACTGCCCTCCATTTGTTTG
**Mosquito UBE4B**	ATCCAGTGATACTGCCGTCC	GGTATGAGCATGTCCTCCGT
**Mosquito CDC20**	TAAAGGATTCCTGCCTGTGG	GTTCATTGAGCTTGCGTTGA
**Mosquito IAP**	TCGCCGTCTACCTCTAGCAT	ACGCAGGATACCAGATGACC
**Mosquito UBE2R**	ATCTCGATTCTGCATCCACC	GAGAGAAGGTGTTTGGCTCG
**Mosquito SKP1**	GCTGACCATGTTGAAGCTGA	ATTTGATTCGACATCCCTGC
**Mosquito PIAS**	CAACCTCCACCACCTGTTCT	AAGAATGAGCCCTCCTGGAT
**Mosquito β1**	GAGAATCAAACCGGTGAGGA	AATGTACGAACTGCCCGAAC
**Mosquito β2**	TCTGTGCGTGATCAAGAAGG	CTCTTGCTCTGTAGCACCCC
**Mosquito β5**	CTGCCGATTGTGTGTACTGG	ACTTGTAGCCGGAGTCCAGA
