# Supplementary material for: Production of Infectious Dengue Virus in Aedes aegypti Is Dependent on the Ubiquitin Proteasome Pathway
Source: PLoS Negl Trop Dis. 2015 Nov 13;9(11):e0004227. doi: 10.1371/journal.pntd.0004227 (PMC4643912; doi:10.1371/journal.pntd.0004227)
Supplement: S3 Table — (PDF) [file pntd.0004227.s006.pdf]

S3 Table. Primers for RNAi assays.

Gene	Forward Primer (5' to 3')	Reverse Primer (5' to 3')
**dsUBE2A**	*TAATACGACTCACTATAGGG*CTTCCTGAACAGCTCCAAGG	*TAATACGACTCACTATAGGG*CGATGGGAAAGTGAAAGGAA
**dsDDB1**	*TAATACGACTCACTATAGGG*CGGTCGTCACATCAAAACAC	*TAATACGACTCACTATAGGG*CATTTGGCCTTGACCTTGTT
**dsUBE2M**	*TAATACGACTCACTATAGGG*GAGCTGAATCTGCCCAAGAC	*TAATACGACTCACTATAGGG*CCTCGCCACACTTTTACCAT
**dsUBE4B**	*TAATACGACTCACTATAGGG*CCAGATTCGCTACTCGGAAG	*TAATACGACTCACTATAGGG*AACCGGTTCTTGGTCAACAG
**dsβ1**	*TAATACGACTCACTATAGGG*GGGCAAGTCGGTGTAAACAA	*TAATACGACTCACTATAGGG*AAAACGGCCTTCTTGACAAA
**dsβ2**	*TAATACGACTCACTATAGGG*TTTTGGGCGCAGATACTAGG	*TAATACGACTCACTATAGGG*TAAGCGTACGCACACACTCC
**dsβ5**	*TAATACGACTCACTATAGGG*CCTTGCTGATGTGTGTGGTC	*TAATACGACTCACTATAGGG*ACTTGTAGCCGGAGTCCAGA
